# What do they need to know: achieving consensus on paediatric musculoskeletal content for medical students

**DOI:** 10.1186/s12909-015-0449-4

**Published:** 2015-10-08

**Authors:** Sharmila Jandial, Jane Stewart, Helen E. Foster

**Affiliations:** 1 Department of Paediatric Rheumatology, Great North Children’s Hospital, Newcastle, UK; 2 School of Medical Education, Newcastle University, Newcastle, UK; 3 Institute of Cellular Medicine, Newcastle University, Newcastle, UK

**Keywords:** Paediatric, Musculoskeletal, Education, Clinical skills, pGALS

## Abstract

**Background:**

Children present commonly with musculoskeletal (MSK) problems, due to a spectrum of causes including potentially life threatening disease, to doctors in varied health care settings. However, doctors involved in the care of children report a lack of confidence in their paediatric musculoskeletal (pMSK) clinical skills and many have little exposure to pMSK teaching. There is no current guidance on the pMSK clinical skills and knowledge required for medical students. The objective of this study was to achieve consensus amongst experts on the learning outcomes for a pMSK curriculum for medical students.

**Methods:**

This was a two-phase study. In Phase one, pMSK educational topics and categories were identified from UK medical students and experts (recruited from pMSK medicine, child health, education and primary care) utilising focus groups and interviews. These themes and concepts informed the structure of learning outcomes that were presented to a Delphi panel in Phase two, with the aim of achieving consensus on the final content of the curriculum.

**Results:**

In Phase 1 participants identified pMSK skills, knowledge and attitudes relevant for medical students. This content was translated into learning outcomes. In Phase 2, the proposed outcomes were submitted to scrutiny by a two-iteration Delphi process with experts in the field. The agreed learning outcomes (*n* = 45) were either generic to child health or specific to pMSK medicine, and related to history taking and examination, knowledge about normal development, key clinical presentation and conditions, approaches to investigation and referral pathways.

**Discussion:**

This study has identified evidence and consensu based content for a pMSK curriculum for medical students, derived from key stakeholders and to be integrated into medical student pMSK teaching.

**Conclusion:**

It is envisaged that implementation of this content will equip graduating doctors with relevant and important skills and knowledge to assess children with MSK presentations, and facilitate early diagnosis and referral to specialist care.

## Background

Musculoskeletal (MSK) problems in childhood are common, accounting for 1 in 8 healthcare visits [1] and a significant source of concern for parents and young people [2]. MSK presentations, such as limp, have a wide differential of causes, ranging from self-limiting trauma to serious illness such as infection or malignancy [3]. In many healthcare systems, children and young people (CYP) will present to doctors who are not specialists in paediatric MSK [pMSK] medicine (such as primary care or emergency medicine); such doctors have responsibility for diagnosis, management and on-going specialist referral.

Appropriate management relies on accurate clinical assessment by the assessing healthcare professional. However delay in access to specialist care is reported in many conditions with MSK presentations including Juvenile Idiopathic Arthritis (JIA) [4–6], cancers [7, 8], muscular dystrophy [9] and slipped upper femoral epiphysis [10]. CYP with MSK problems invariably do not present to specialists directly, referral pathways are often complex and reasons for delay are multifactorial [11]. Delay at physician level is likely to include sub-optimal pMSK clinical skills; doctors involved in the care of CYP report low self-confidence in their pMSK clinical skills [12–15], demonstrate poor performance in clinical practice [16] and have little recall of pMSK teaching at undergraduate or postgraduate level [17]. These observations are not surprising given that pMSK teaching is infrequently included within adult MSK or child health teaching in both the UK and US [18–20]. Furthermore despite consensus that pMSK clinical skills are as important as other bodily systems, they are less well taught within child health in the medical student curriculum [17].

Several initiatives have developed to improve the profile of MSK education. The Gait, Arms, Legs and Spine screening examination (GALS) [21] and Regional Examination of the Musculoskeletal System (REMS) [22] are now routinely taught at UK medical schools [18, 23] and have been shown to improve doctors and medical students’ confidence and performance in the assessment of the adult MSK system [23–25]. The paediatric version of GALS, called pGALS, has been developed as a simple, rapid pMSK assessment [26]; pGALS has been validated in school-aged children [27], shown to be practical and effective in clinical practice [28, 29] and is supplemented by the paediatric version of REMS (pREMS) although this is primarily aimed at postgraduate training [30].

These structured and evidence based tools aim to improve MSK clinical skills although as educational interventions, their potential is likely to be optimised when taught with essential knowledge about disease and clinical presentations. In the context of CYP, this knowledge must include understanding of normal development and differences from adult practice. Medical school content is often ‘outcome-based’ [31] where the curriculum is driven by the outcomes that students should achieve. Learning outcomes (LOs), provide the framework from which the rest of the curriculum (such as environment, assessment, evaluation) can be developed [32].

To date, there are no LOs for pMSK clinical skills and knowledge required at medical student level. The recommended global curriculum for undergraduate MSK medicine [33], subsequently further developed for postgraduate education [34], is focussed primarily on adults but includes the paediatric themes of fractures, JIA, infections and hip disorders. The US curriculum for paediatrics [35] refers to pMSK clinical skills. There are descriptions of normal variants of MSK posture [36] and how pMSK clinical assessment compares to adults [37] but not within LOs.

Our study aimed to establish pMSK LOs for medical students encompassing the spectrum of pMSK medicine (both rheumatology and orthopaedics) and using healthcare educational research methods. We envisage that such pMSK learning outcomes would set the basic clinical skills and knowledge for all graduating doctors, irrespective of their subsequent career pathway.

## Methods

The intent of the methods chosen in this study was to identify an agreed set of LOs from a mixed group of specialists across the UK.

Consensus methods have the aim of canvassing opinion from a panel of experts; this is particularly relevant in pMSK medicine where the views of primary care doctors, paediatricians and pMSK specialists are all required. The modified Delphi method is used commonly within healthcare research [38–40] with consensus achieved from opinions of a panel of experts using an iterative approach [41]. Results from each iteration are collated and fed back to participants in the next round(s) in order to give ‘controlled feedback’, enabling them to compare their responses with other panel members in a structured and objective way. While the gold standard for consensus is 100 % agreement from panel members, a level is normally set prior to conducting the research [42, 43]. In healthcare research this is often set at 75 or 80 % [40, 42].

**Fig. 1:**
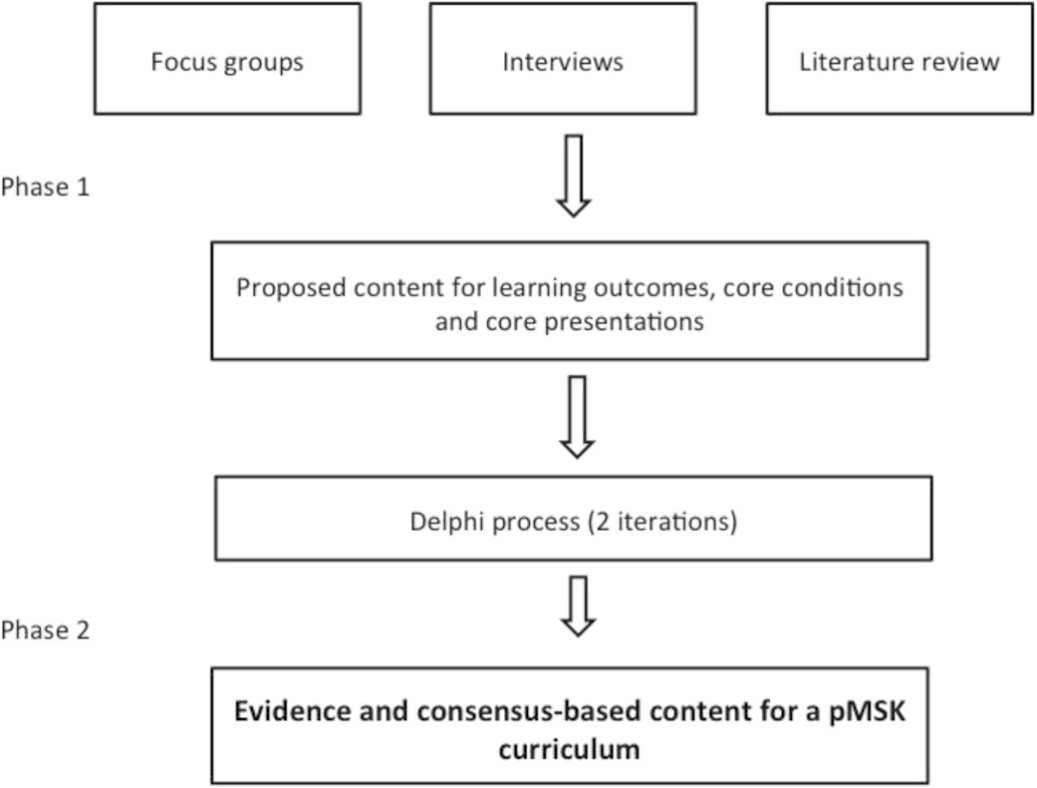
Overview of methodology

A two-phase approach (Fig. 1) was chosen for this study. Phase 1 had the aim of generating knowledge, skills and attitudes within pMSK education to be included within a modified Delphi process in Phase 2. This study was given full ethical approval by the Newcastle and North Tyneside 1 Research Ethics Committee (MREC No 07/H0906/101).

Key stakeholder groups were identified at the outset of this study through the researchers’ prior work in this area [4] in order to ensure representation in both phases of the study. Stakeholder groups represented the different environments in which CYP may present (primary care, paediatrics, pMSK specialists (rheumatology and orthopaedics)). In addition, to ensure the final outputs were fit for purpose, medical educators and teachers were included. These groups were deemed pMSK education stakeholder groups. Medical students were involved in Phase 1 to give insight into important components of their current curriculum and what would, from their perspective, inform a model for pMSK teaching in terms of concepts and format; we deemed this important, as students are the ultimate recipients of curricula design. Students were recruited from three UK medical schools in order to ensure a geographical mix. Given the focus on knowledge, it was deemed inappropriate to involve patients and families in the research.

Clinical practice experts were identified from their membership of professional bodies, namely British Society of Paediatric and Adolescent Rheumatology, British Society for Children’s Orthopaedic Surgery, Association for the Study of Medical Education, and Primary Care Rheumatology Society. Stakeholders were invited to participate in the Delphi process through their links with professional bodies and purposive sampling was used to ensure UK-wide representation from all groups [44].

The aim of Phase 1 was to generate ideal content and themes within pMSK education for medical students. A topic guide, informed by the available literature, provided structure to focus groups, facilitated by a sole researcher (SJ). Individual interviews allowed in-depth discussion of controversial areas and could be targeted to under-represented stakeholder groups. All interviews and focus groups were audio-recorded with participants’ consent. Transcripts were reviewed by the research team and framework analysis [45] used to identify topics and categories which framed the emergent data.

The aim of phase 2 was to achieve consensus on the proposed pMSK LOs, core conditions and core presentations. Medical students were excluded from this stage, as they were deemed ‘non-expert’ in this context. Two rounds of the Delphi process were conducted. Prior to the first round, personalised invitations were sent to all participants and consent obtained. Data sheets could be returned by email, fax or post. An email reminder was sent at 2 and 4 weeks following both rounds. In the first round, participants were asked to ‘accept’, ‘reject’ or ‘modify’ LO statements, with space for free-text comments. Round 1 data sheets were analysed by the research team to assess agreement levels and free text comments. Quantitative data was analysed using SPSS [46] and framework analysis used to review qualitative data [45]. Results informed content for the second data sheet, in which participants were asked to only ‘accept’ or ‘reject’ the modified statements. A consensus level of 80 % at the end of Round 2 was agreed by the research team prior to the Delphi process. From phases 1 and 2, a final set of pMSK LOs were collated with *generic* content integral to general paediatrics and those *specific* for pMSK medicine. In addition the final list of core presentations and core conditions were proposed.

## Results

### Participants

Representation of pMSK stakeholder groups was achieved at all stages in this study. In Phase 1, focus groups with healthcare professionals were held alongside professional meetings of UK paediatric rheumatologists and orthopaedic surgeons. Two further focus groups had mixed representation from paediatrics, subspecialties and primary care, including child health teachers. Individual interviews (*n* = 4) were held with orthopaedic surgeons, educationalists and paediatric oncologists. In addition, further focus groups (*n* = 3) were held with medical students who were at the end of their child health rotations at three UK medical schools: Glasgow, Newcastle and Birmingham. In phase 1, participants involved were medical students *n* = 18; paediatricians *n* = 9; primary care *n* = 1; educationalist *n* = 2; paediatric rheumatology *n* = 8; paediatric orthopaedics *n* = 3; paediatric emergency medicine *n* = 1.

The Delphi process involved 35 participants with representation from paediatric orthopaedics (5/35, 14 %), paediatric rheumatology (7/35, 20 %), general paediatricians with rheumatology interest (3/35, 9 %), general paediatricians with other interest (5/35, 14 %) educationalists (7/35, 20 %) and primary care (8/35, 23 %).

### Phase 1

Qualitative data generated in Phase 1 underwent framework analysis [45]. Emergent topics led to categorisation of data including specific diseases that medical students should be aware of (‘core conditions’) [*n* = 6] and knowledge of the different ways in which CYP with pMSK diseases may present (‘core presentations’) [*n* = 14]. Understanding of ‘red flags’ as presenting features of life threatening conditions such as malignancy, infection and non-accidental injury recurred as themes. Students and experts proposed broad themes within child health relating to items of knowledge, skills and attitudes [*n* = 33] within categories of ‘History taking’, ‘Examination’, ‘Investigations’, and ‘Management’. Expert opinion further divided these items into specific and objective LOs [*n* = 51] [47]. In addition, students and teachers both offered suggestions on how content could be delivered and taught to inform a final curriculum.

### Phase 2

The final curriculum content (in the form of LOs, core conditions and core presentations) is listed in Tables 1 and 2. Response rate for the Delphi process was 33/35 in Round 1 and 34/35 in Round 2 with participants from stakeholder groups as listed above. Suggested modifications and new content suggested by the Delphi panel led to 10 new LOs being generated and included within the Round 2 data sheet. Only LOs with high consensus (>97 %), and without modifications proposed were accepted into the final curriculum after Round 1. With the exception of ‘recognise the pMSK presentations of malignancy’, these LOs were deemed to be generic to skills required by a medical student. Following Round 2 of the Delphi process, all statements with 80 % agreement or above were deemed to be included in the curriculum.

**Table 1:** Core conditions and presentations

Core conditions	Core presentations
Juvenile idiopathic arthritis	Swollen joint (s)
Septic arthritis & osteomyelitis	A limp
Paediatric hip disorders (Developmental Dysplasia of the Hip, Slipped Upper Femoral Epiphysis, Legg-Calve-Perthé disease)	A fracture
Reactive arthritis	An unexplained fever
Bone & Joint malignancy	Loss of function
Normal variants	Joint or back pain
Talipes equinovarus	
Nocturnal idiopathic pain (growing pains)	
Common fractures e.g. forearm and multiple fractures including non-accidental Injury

**Table 2:** Learning Outcomes for pMSK medicine for medical students

Generic Child Health Learning Outcomes
Establishing interaction
1. Establish rapport with child and family.
2. Respect privacy and confidentiality for the child and family.
3. Use appropriate behaviour and language in relation to the developmental stage of the child.
4. Modify history taking and examination according to child’s developmental stage (e.g. questions about functional activities).
History Taking
5. Recognise symptoms such as persistent pain, night pain, fever and weight loss as red flag symptoms for malignancy or significant systemic disease.
6. Elicit and document a pain history.
7. Identify major milestones within development.
8. Use a pain score or simple tools to assess level of pain.
Examination
9. Demonstrate an understanding of ways to engage children when examining to maintain co-operation and minimise discomfort.
10. Demonstrate awareness of developmental staging.
11. Demonstrate awareness that a neurological examination may be indicated (e.g. in the context of back pain) and the important associations such as paraesthesiae and loss of bladder/bowel function.
Investigations
12. Identify the role of blood tests such as FBC, ESR, CRP.
13. Discuss the indications for plain X-ray.
14. Demonstrate a systematic approach to interpretation of plain X-rays (e.g. of bony fracture).
15. Discuss the purpose of other investigations such as CT (to look at bone), MRI (to look at soft tissue) or bone scan (to look for inflammatory disease such as bony metastases or osteomyelitis).
Management
16. Summarise key points in the history and examination to form an overall impression.
17. Use appropriate medical terminology in discussion with professional colleagues including anatomical landmarks where appropriate (e.g. extensor, flexor surfaces, relation to bones, muscles or joints).
18. Relate history and examination findings to core conditions.
19. Formulate a provisional differential diagnosis for core presentations.
20. Demonstrate a structured ‘surgical sieve’ approach to a differential diagnosis (e.g. timing, possible aetiology such as inflammatory, infective, malignancy).
21. Communicate provisional proposed management plan verbally to child and family after discussion with their teachers.
22. Demonstrate awareness of the importance of a multi-disciplinary team in managing a child with musculoskeletal disease.
23. Outline the principles of managing children with chronic disease (e.g. considering impact on school, play and family, need for medications and monitoring, and the role of healthcare professionals).
24. Plan and discuss a simple approach to the management of pain - use of a pain ladder, reassurance and simple analgesia
25. Help medical staff in liaising with other healthcare providers regarding management plan e.g. nursing staff, primary care, physiotherapist.
pMSK specific learning outcomes
History taking
26. Record pattern of injury.
27. Demonstrate awareness of injury patterns suggestive of Non-Accidental Injury.
28. Recognise the importance of a full family and social history and their relevance to musculoskeletal presentations.
29. Recognise the need for extended musculoskeletal history in certain presentations (e.g. limp, pain, rashes, refusing to walk).
30. Include a brief musculoskeletal history in review of systems in all history taking encounters.
31. Recognise features in the history that may distinguish mechanical from inflammatory musculoskeletal pathology.
Examination
32. Perform an examination that screens the musculoskeletal system (e.g. paediatric Gait, Arms, Legs, Spine) understanding that positive findings should lead to more detailed examination.
33. Demonstrate the principles of regional musculoskeletal examination incorporating a look, feel, move approach.
34. Demonstrate awareness that limitation of movement of joints could arise from pathology within the joint, muscle or bone.
35. Recognise that skin and nail abnormalities may be associated with musculoskeletal disease (e.g. nail pitting, rashes).
36. Identify clinical features that suggest an inflamed joint.
37. Recognise clinical features suggestive of a septic joint and the place of appropriate investigations and referral.
38. Recognise that normal children have increased joint flexibility compared to adults and may be hypermobile.
39. Recognise that Marfan’s and Ehler’s Danlos syndromes may be associated with hypermobility.
40. Observe and describe principles of gait patterns (e.g. symmetry, leg alignment, presence of pain, limp).
41. Demonstrate awareness that leg alignment and foot posture changes with age and normal variants within these - knock knees, bow legs, flat feet, in-toeing.
42. Elicit signs of muscle weakness and be aware of the possibility of proximal myopathy.
Investigations
43. Discuss results of FBC, ESR, CRP in context of musculoskeletal presentations and potential implications (e.g. raised white cell count and possible sepsis).
Management
44. Describe musculoskeletal presentations of malignancy such as nocturnal bone pain, swelling, systemic features such as weight loss.
45. List specialist opinions that may be necessary for musculoskeletal conditions (e.g. orthopaedics, rheumatology, ophthalmology) and discuss when this may be relevant.

The final curriculum included LOs (*n* = 45), alongside core presentations (*n* = 6), and core conditions (*n* = 9) (Table 1) to provide context and to aid understanding. LOs were within the domains of ‘establishing interaction’ (with child/carer) [*n* = 4], ‘history taking’ [*n* = 10], ‘physical examination’ [*n* = 14],’ initial investigations’ [*n* = 5] and ‘management’ [*n* = 12]. Generic child health skills and attitudes (LOs *n* = 25) recur throughout the curriculum such as awareness of normal development, communication skills and awareness of safeguarding. pMSK specific LOs (*n* = 20) related mainly to history taking (*n* = 6) and examination (*n* = 11). The full list of LOs, separated into generic and pMSK specific, is listed in Table 2.

## Discussion

This study has developed, to our knowledge, the first evidence and expert consensus-based pMSK LOs, core conditions and core presentations for medical students to achieve by the time of graduation. This is important, as implementation of this curriculum will embed core pMSK skills and knowledge at a time when essential clinical skills are acquired. Skills and knowledge can then be developed further during postgraduate experience and regardless of the eventual career path.

In the UK, this pMSK curriculum has particular relevance as newly graduated doctors will rotate between specialties for the first two years of their postgraduate career [48]; many, but not all, will work in primary care, accident and emergency and paediatrics, and be responsible for the care of CYP at the initial stage of clinical presentations. It is noteworthy that in the UK, primary care doctors may not work within paediatrics during their vocational training [49], thereby making the inclusion of pMSK content in their undergraduate child health education, of even greater importance so that they have basic skills and knowledge.

Methods used in this study have ensured that the final content reflects the views and consensus of multiple stakeholders, including primary care and paediatrics, as opposed to previous work, which included only the opinions of MSK specialists [33]. By engaging a breadth of stakeholders at primary, secondary and tertiary level as well as educationalists, this curriculum reflects the cross-cutting themes and level of skill and knowledge to be acquired by medical students at the time of graduation. Consensus methods allowed agreement to be achieved from a UK-wide expert panel; similar methods were used to develop undergraduate curricula in psychiatry [38], anaesthetics [40] and dermatology [39]. The inclusion of medical students enabled their opinion to link pMSK teaching with existing child health teaching.

There are 45 LOs (Table 2), with the majority being generic to child health, although relevant to the child with a pMSK presentation, and a fewer number (*n* = 20) being specific to pMSK medicine. Generic LOs include communication skills with CYP at varying ages, assessment of pain and knowledge of normal pMSK development, as well as awareness of ‘red flag’ presenting features of serious disease with MSK features such as malignancy, infection, and non-accidental injury. We would argue that it is difficult to develop a pMSK curriculum without generic content, reflecting complex clinical encounters which are not uncommon in paediatrics. The specific pMSK LOs included common conditions within primary care and paediatrics such as minor trauma, alongside indicators of important conditions within rheumatology and orthopaedics (such as inflammatory arthritis or hip disorders). Investigations and management LOs included rationale and interpretation of baseline investigations, the importance of communication with professional colleagues and awareness of referral pathways to MSK specialists. The level of knowledge and skills required are reflected in the agreed LOs; for example pGALS was agreed to be the appropriate level of examination skill but with awareness of the principles of more detailed MSK examination based on ‘look, feel, move, measure, function’ as used in pREMS [30].

The list of core conditions can be mapped to core presentations (Table 1) to provide the teacher and student context upon which teaching and learning can be based. For example, the ‘limping child’ as a core presentation is ideal, as a common presentation to primary and secondary care, with a wide differential diagnosis [3]. The approach to diagnosis encompasses many of the LOs, both generic and specific (potentially 30 of the proposed LOs), including “red flags”, and 7 of the 14 core conditions.

There are limitations to our study. Firstly, the project involved UK stakeholders and was focussed on UK curriculum requirements, although we anticipate that the essential themes and concepts are applicable to medical schools elsewhere. The consensus methods used allow opinion of a relatively small number of experts although we addressed this through recruitment of representatives from different specialties and different hospitals across the UK. Paediatric emergency clinicians were not involved but doctors involved in acute paediatrics and orthopaedics did take part. pMSK medicine can be seen as a specialist field, but by involving primary care doctors and general paediatricians we have included the generalist view to ensure that the scope of the LOs are appropriate in content and level for the graduating doctor. A limitation of consensus methods relates to the level of agreement, and the point at which consensus is deemed to have been achieved; this study used a cut off set at 80 %. This is consistent with pragmatic approaches to agreeing consensus amongst experts.

The number of pMSK LOs may be deemed a barrier to implementation given the increasing pressures on medical schools to address the ever-changing field of medicine. However the LOs are based on generic themes and can be mapped easily to a child health and clinical skills curriculum. It is envisaged that the pMSK LOs will be taught within child health with teachers drawn from general paediatrics and primary care; such teachers are unlikely to be pMSK specialists and may need additional resources and support.

## Conclusions

This study proposes pMSK LOs to be achieved by medical students by the time of graduation. Consensus methodology has ensured that views from all pMSK stakeholders, including generalists and specialists, have been represented. Clinical skills and knowledge are key, reflect the needs of doctors involved in the care of CYP and include history taking and examination, recognition of serious illness and awareness of common presentations and conditions presenting in childhood.

It is anticipated that doctors who learn these pMSK LOs, will be equipped with the essential clinical skills and knowledge to assess children with MSK presentations in an appropriate and effective manner. With time, it is envisaged that all doctors involved in the care of CYP will have improved performance of pMSK assessment with the ultimate aim of earlier recognition of significant disease and prompt referral to specialist care as appropriate to facilitate improved clinical outcomes.

## Abbreviations

MSK: Musculoskeletal
pMSK: paediatric Musculoskeletal
CYP: Children and Young People
GALS: Gait, Arms, Legs and Spine
pGALS: paediatric Gait, Arms, Legs and Spine
REMS: Regional Examination of the Musculoskeletal System
pREMS: paediatric Regional Examination of the Musculoskeletal System
LO: Learning Outcome
